# A systematic review of randomisation method use in RCTs and association of trial design characteristics with method selection

**DOI:** 10.1186/s12874-022-01786-4

**Published:** 2022-12-07

**Authors:** Cydney L. Bruce, Edmund Juszczak, Reuben Ogollah, Christopher Partlett, Alan Montgomery

**Affiliations:** Nottingham Clinical Trials Unit, University of Nottingham, Applied Health Research Building, Nottingham, NG7 2RD UK

**Keywords:** Randomisation, Stratification, Minimisation, Allocation, Review

## Abstract

**Background:**

When conducting a randomised controlled trial, there exist many different methods to allocate participants, and a vast array of evidence-based opinions on which methods are the most effective at doing this, leading to differing use of these methods. There is also evidence that study characteristics affect the performance of these methods, but it is unknown whether the study design affects researchers’ decision when choosing a method.

**Methods:**

We conducted a review of papers published in five journals in 2019 to assess which randomisation methods are most commonly being used, as well as identifying which aspects of study design, if any, are associated with the choice of randomisation method. Randomisation methodology use was compared with a similar review conducted in 2014.

**Results:**

The most used randomisation method in this review is block stratification used in 162/330 trials. A combination of simple, randomisation, block randomisation, stratification and minimisation make up 318/330 trials, with only a small number of more novel methods being used, although this number has increased marginally since 2014. More complex methods such as stratification and minimisation seem to be used in larger multicentre studies.

**Conclusions:**

Within this review, most methods used can be classified using a combination of simple, block stratification and minimisation, suggesting that there is not much if any increase in the uptake of newer more novel methods. There seems to be a noticeable polarisation of method use, with an increase in the use of simple methods, but an increase in the complexity of more complex methods, with greater numbers of variables included in the analysis, and a greater number of strata.

**Supplementary Information:**

The online version contains supplementary material available at 10.1186/s12874-022-01786-4.

## Background

Randomisation is an important feature in clinical trials; it allows valid estimation of standard errors, is vital to conceal allocations and maintain this concealment in blinded trials and allowing researchers to eliminate important sources of bias. For example, selection bias is introduced when recruiters assign participants to certain treatments leading to group imbalances. Chronological bias, caused by imbalances in the timing of the assignment of treatments over the recruitment period, can also lead to group differences. Randomisation, implemented correctly, mitigates the risk that the study findings could be influenced by these and other unknown sources of bias. Simple randomisation creates entirely unpredictable random sequences, whereas methods such as block and stratified randomisation are designed to generate more balanced groups with respect to group size and population characteristics, potentially at the cost of more predictable sequences.

Each method may perform differently depending on the design of the trial and there is a lack of consensus on which methods are most appropriate in different situations. For example, simple randomisation can be highly effective in large trials but is not advised if a trials target sample size is less than 200 participants to avoid potential imbalances in treatment group size, which can affect the power of subsequent analysis [1]. Minimisation is considered by some as “the platinum standard” of randomisation methods for achieving balanced groups, whilst others strongly discourage the use of dynamic methods for this purpose [2, 3], regarding less complex methods such as simple or block randomisation as more effective [4].

It has been suggested that choice of allocation method should be dependent on the study context, objectives, and resources [5]. It was also stated that simpler methods are preferred, and that the size of the study was a key factor in selecting a randomisation method. Previous reviews have described the association between randomisation methodology and sample size [4], and changes in reporting before and after CONSORT guidance updates [6].

The aim of this study is to describe current randomisation methods use, investigate associations between choice of method and trial population characteristics, and the extent to which method selection has changed over time.

## Methods

This review looks at the randomisation methods used in trials reported in five high impact journals. The BMJ, NEJM, JAMA and The Lancet were identified to allow comparisons with previous similar reviews [4, 6]. The NIHR HTA library was added as an additional journal to widen the scope of this review.

Papers from the four clinical journals were identified from the database PubMed. This search was defined as papers where the title and/or abstract contained the words “RCT”, “randomised” or “randomized”, published from January 2019 up to the end of December 2019.

**Fig. 1:**
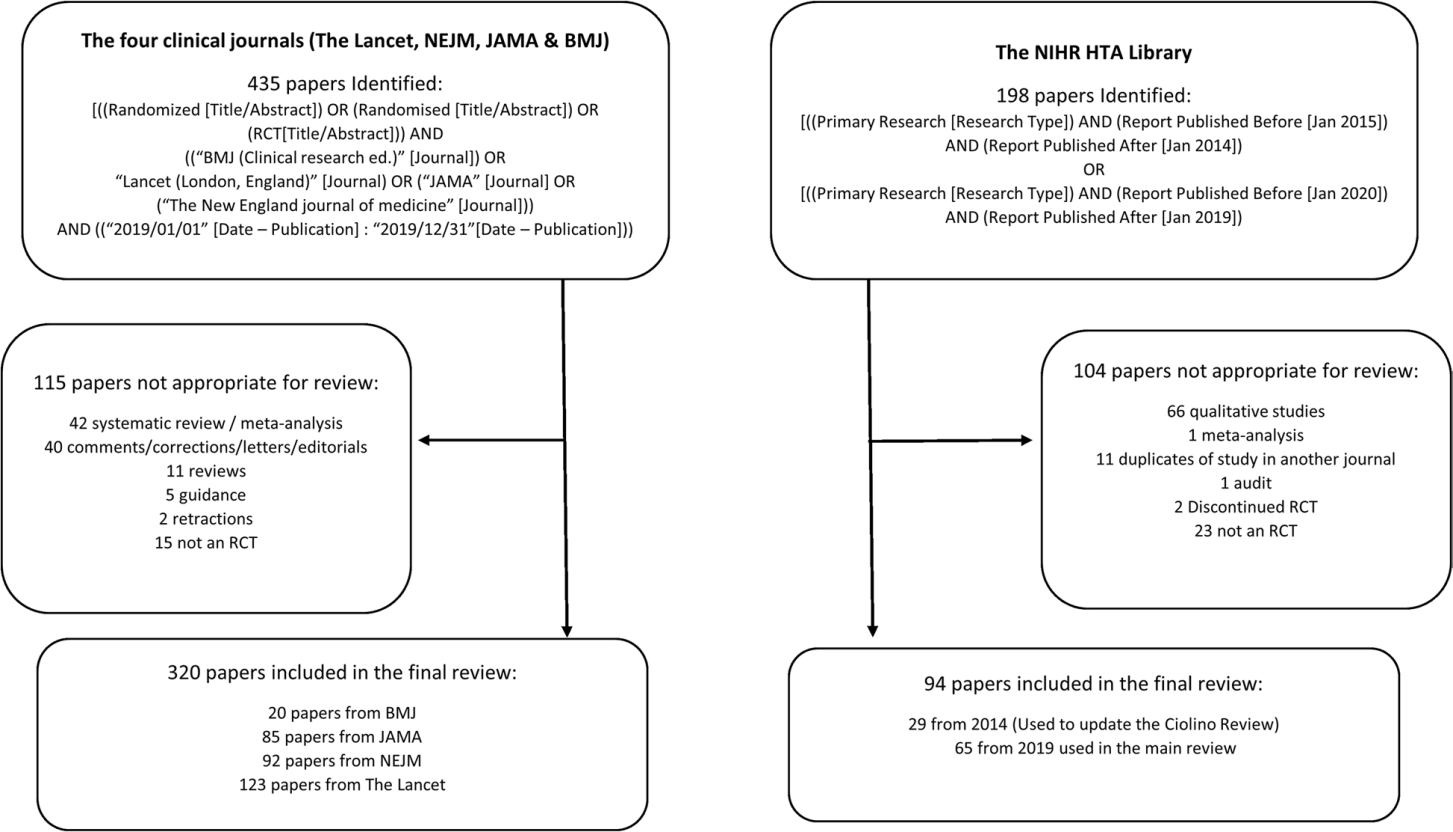
Article search results including identified papers, reasons for exclusions and final numbers of papers included

The NIHR HTA library was searched to identify journal reports published from January 2019 up to the end of December 2019 (and January 2014 to the end of December 2014) categorised as primary research. For each article, we verified that the study had not already been published in one of the other four journals. This was also done for the 2014 extraction, comparing if these fit the search criteria originally used. A full breakdown of this search criteria, along with reasons for exclusions and final numbers is given in Fig. 1.

Each paper was reviewed to collect information on the randomisation method used and trial design features such as sample size, number of arms and centres, blinding methods used and details of variables used in the randomisation (see Additional file 2: Appendix Table 1 for full details of the data extraction). If this information could not be found in the paper this was recorded, and then other relevant sources were searched; the protocol, statistical analysis plan or other related publications. If the randomisation method could not be retrieved the author was contacted to obtain this information. Data was only recorded as unknown if it could not be verified in any of these sources.

**Table 1:** Summary table showing the main study characteristics of 2019 papers split by randomisation method

Study characteristic		Total [n (%)]	Simple [n (%)]	Block [n (%)]	Stratified [n (%)]	Block Stratified [n (%)]	Minimisation [n (%)]
Unit of Allocation	Individual	348 (90)					
	Cluster	37 (10)					
If Cluster, Number of Clusters	2–25	15 (40)					
	26–50	8 (22)					
	51 +	14 (38)					
**For individually randomised studies** ^a^							
		**(*n* = 330)**	**(*n* = 20)**	**(*n* = 33)**	**(*n* = 66)**	**(*n* = 162)**	**(*n* = 49)**
Number of Arms	2	270 (82)	16 (80)	30 (91)	51 (77)	128 (79)	45 (92)
	3	39 (12)	3 (15)	1 (3)	14 (21)	18 (11)	3 (6)
	4	15 (4)	0	1 (3)	1 (2)	12 (7)	1 (2)
	5 +	6 (2)	1 (5)	1 (3)	0	4 (3)	0
Number of Centres ^b^	Single Centre	27 (8)	4 (20)	5 (15)	4 (6)	13 (8)	1 (2)
	2–10	81 (25)	4 (20)	11 (34)	10 (15)	51 (31)	5 (10)
	11–25	65 (20)	7 (35)	4 (12)	4 (6)	40 (25)	10 (20)
	26–50	45 (14)	1 (5)	4 (12)	13 (20)	13 (8)	14 (29)
	51–100	39 (12)	1 (5)	4 (12)	11 (17)	15 (9)	8 (16)
	101 +	70 (21)	3 (15)	5 (15)	24 (36)	27 (17)	11 (23)
	**Median (IQR)**	**22 (7, 86)**	**15 (4, 29)**	**11 (4, 51)**	**61 (17, 153)**	**16 (6, 53)**	**42 (14, 90)**
	**Mean (SD)**	**72 (144)**	**59 (127)**	**121 (324)**	**111 (137)**	**51 (97)**	**62 (64)**
Size of Study	< 200	60 (18)	6 (30)	10 (30)	15 (23)	23 (14)	5 (10)
	201–1000	159 (48)	9 (45)	15 (46)	26 (39)	86 (53)	24 (49)
	1001–10,000	103 (31)	4 (20)	6 (18)	24 (36)	49 (30)	20 (41)
	10,001 +	8 (3)	1 (5)	2 (6)	1 (2)	4 (3)	0
	**Median (IQR)**	**648 (241, 1468)**	**354 (133, 1445)**	**417 (154, 782)**	**690 (225, 1672)**	**653 (253, 1326)**	**694 (334, 2135)**
	**Mean (SD)**	**1702 (3835)**	**1493 (2910)**	**1845 (3980)**	**1844 (3275)**	**1748 (4581)**	**1346 (1343)**
Blinding	No	194 (59)	9 (45)	18 (55)	19 (29)	113 (70)	35 (71)
	Yes	136 (41)	11 (55)	15 (45)	47 (71)	49 (30)	14 (29)

Unless otherwise specified, the table reports the number of studies along with column percentages

^a^ 6 trials that used stratified minimisation and 12 defined as other are not included

^b^ 3 trials that used block stratification did not report the number of centres

Validation was performed at two stages. A random sample of 20 papers deemed ineligible for review were checked to confirm agreement on the inclusion criteria. Then, a random sample of 24 papers were independently extracted by another reviewer. Separate definitions were given for disagreements and discrepancies (see Additional file 3: Appendix Table 2 for full definition). Discrepancies covered any discordant items, and all discrepancies found were resolved by consensus. We decided that if the number of disagreements was greater than 5%, then a further sample would be examined.

**Table 2:** Comparison of inclusion of randomisation variables in subsequent analysis for the different randomisation methods

Study Characteristics		No covariates in analysis (*n* = 71)	A covariate included in analysis (*n* = 212)	Total
Allocation method ^a^	No randomisation covariates (Simple/Block)	-	-	53
	Stratified (Centre only)	32 (45)	26 (12)	58
	Stratified	30 (43)	140 (66)	170
	Minimised	8 (11)	41 (19)	49
	Stratified and Minimised	1 (1)	5 (3)	6

Unless otherwise specified, the table reports the number of studies along with column percentages

^a^ 12 trials where the randomisation method is defined as other are not included

Trial characteristics and choice of randomisation method were compared with the 2014 Ciolino et al. review [6]. The use of stratification or minimisation methods to balance allocation, the type of variables used in balancing, and inclusion of such variables in analysis were compared with a 2010 review by Kahan et al. [7] In addition, where randomisation balanced for recruiting site or centre, the method of any subsequent adjustment in the analysis was also explored.

## Results

For the random sample of 20 excluded papers, there was 100% agreement between reviewers. For the full data extraction review, 10 discrepancies were found across 24 items in 26 papers, meaning the proportion of discrepancies fell below our prespecified 5% threshold at 1.1%.

Our search returned 633 articles of which 414 (65%) were eligible for inclusion. Main reasons for exclusions were that a paper consisted of a meta-analysis or that it was a comment or correction. For the NIHR HTA library, as the only search criteria was primary research in 2019, many of the papers reviewed contained qualitative research (see Fig. 1 for a breakdown of reasons for exclusions).

The most commonly used randomisation method in 2019 in individually randomised trials was block stratified randomisation in 162/330 trials (47%). Almost two thirds 228/330 used some form of stratification within their randomisation. All but 12 of the randomisation methods used can be classified as using simple, block, stratification minimisation or a combination of these.

Within this review, 348 of the trials were individually randomised, and 37 were cluster randomised. For the cluster randomised trials, 25 of these trials can be classified using the above methods (again with the most common being stratification) and additional methods used included step wedged randomisation, matched pairs of clusters and allocation of clusters to minimise imbalance.

One trial began using block stratification and during recruitment detected imbalances in groups and so switched their allocation method to minimisation. In this case we have classified this randomisation as block stratification as this was the intended randomisation method at the design phase.

Of the 12 randomisation methods that are classified as other, 3 of these used Bayesian methods, 4 defined their methods as dynamic or adaptive, 1 used a biased coin design, 1 used a big stick design, 1 defined their method as using cohorts and 2 did not properly specify the methods and there was no access to the protocol (and no response from the authors).

Table 1 shows a breakdown of trial population characteristics by randomisation method. We can see that sample size and number of centres relate to randomisation method selection. Interestingly, the number of centres is much larger for studies that used stratification. After filtering out trials where centre was included in the randomisation and those that were not, the median number of centres is still much larger in stratified studies, but not those that included centre as a stratification variable (see Additional file 4: Appendix Table 3).

**Table 3:** The characteristics of each variable used to randomise split by covariate inclusion in the analysis

Variable characteristics		Covariate not in analysis (*n* = 168)	Covariate included in analysis (*n* = 479)
Variable type^a^	Binary	45 (27)	194 (41)
	Categorical (Ordinal)	5 (3)	38 (8)
	Categorical (Non-Ordinal)	100 (59)	126 (26)
	Continuous	18 (11)	121 (25)
Variables form in randomisation if continuous	Binary	9 (50)	90 (74)
	Categorical	5 (28)	26 (22)
	Unknown	4 (22)	5 (4)
Number of categories ^b^	2	59 (35)	304 (64)
	3	16 (9)	64 (13)
	4	10 (6)	20 (4)
	5 +	72 (43)	77 (16)
	Unknown	11 (7)	14 (3)
	**Median (IQR)**	**3 (2, 17)**	**2 (2, 3)**
	**Mean (SD)**	**29 (77)**	**6 (17)**

Unless otherwise specified, the table reports the number of studies along with column percentages

^a^ Variable type refers to the form of the variable separate of how it was included in the randomisation

^b^ Some categorical variables were split as binary to include in the randomisation. This includes 5 covariates not in the analysis and 20 covariates in the analysis

From Table 2, comparing the trials with no covariates in the analysis to those with at least one covariate in the analysis, these trials are much more likely to be stratified by centre, suggesting that generally if a trial is stratified by centre, centre is not subsequently included in the analysis.

Table 3 provides a breakdown by the individual characteristics of the variable. Comparing covariates that were not included in the analysis to those that were, covariates not included in the analysis are less likely to be binary than those included in the analysis and are more likely to be non-ordinal categorical. This may be due to the fact that centre is often not included within the analysis, supported by the fact that covariates not included in the analysis generally had a much greater number of categories.

In Table 4, comparing stratification variables to minimisation variables we see a similar pattern, with a higher percentage of stratification variables being non-ordinal categorical and having more than five categories. To confirm this, we split these tables out to look specifically at centre and those variables that were prognostic. This effect can still be seen even when looking at prognostic variables only suggesting there is another reason for this effect (Table not shown).

**Table 4:** The characteristics of each variable used to randomise split by randomisation method (stratification or minimisation)

Variable Characteristics		Stratified (*n* = 436)	Minimised (*n* = 206)
Variable type^a^	Binary	157 (36)	80 (39)
	Categorical (Ordinal)	20 (5)	22 (11)
	Categorical (Non-Ordinal)	175 (40)	49 (24)
	Continuous	84 (19)	55 (27)
Variables form in randomisation if continuous	Binary	61 (73)	38 (69)
	Categorical	20 (24)	11 (20)
	Unknown	3 (4)	6 (11)
Number of categories ^b^	2	233 (53)	128 (62)
	3	54 (12)	24 (12)
	4	19 (5)	11 (5)
	5 +	115 (26)	33 (16)
	Unknown	15 (4)	10 (5)
	**Median (IQR)**	**2 (2,6)**	**2 (2,3)**
	**Mean (SD)**	**13 (48)**	**10 (29)**

Unless otherwise specified, the table reports the number of studies along with column percentages

^a^ Variable type refers to the form of the variable separate of how it was included in the randomisation. In the minimisation group, 4 variables were classed as “other” so not included

^b^ Some categorical variables were split as binary to include in the randomisation. This includes 15 covariates that were stratified on and 10 covariates that were minimised

**Fig. 2:**
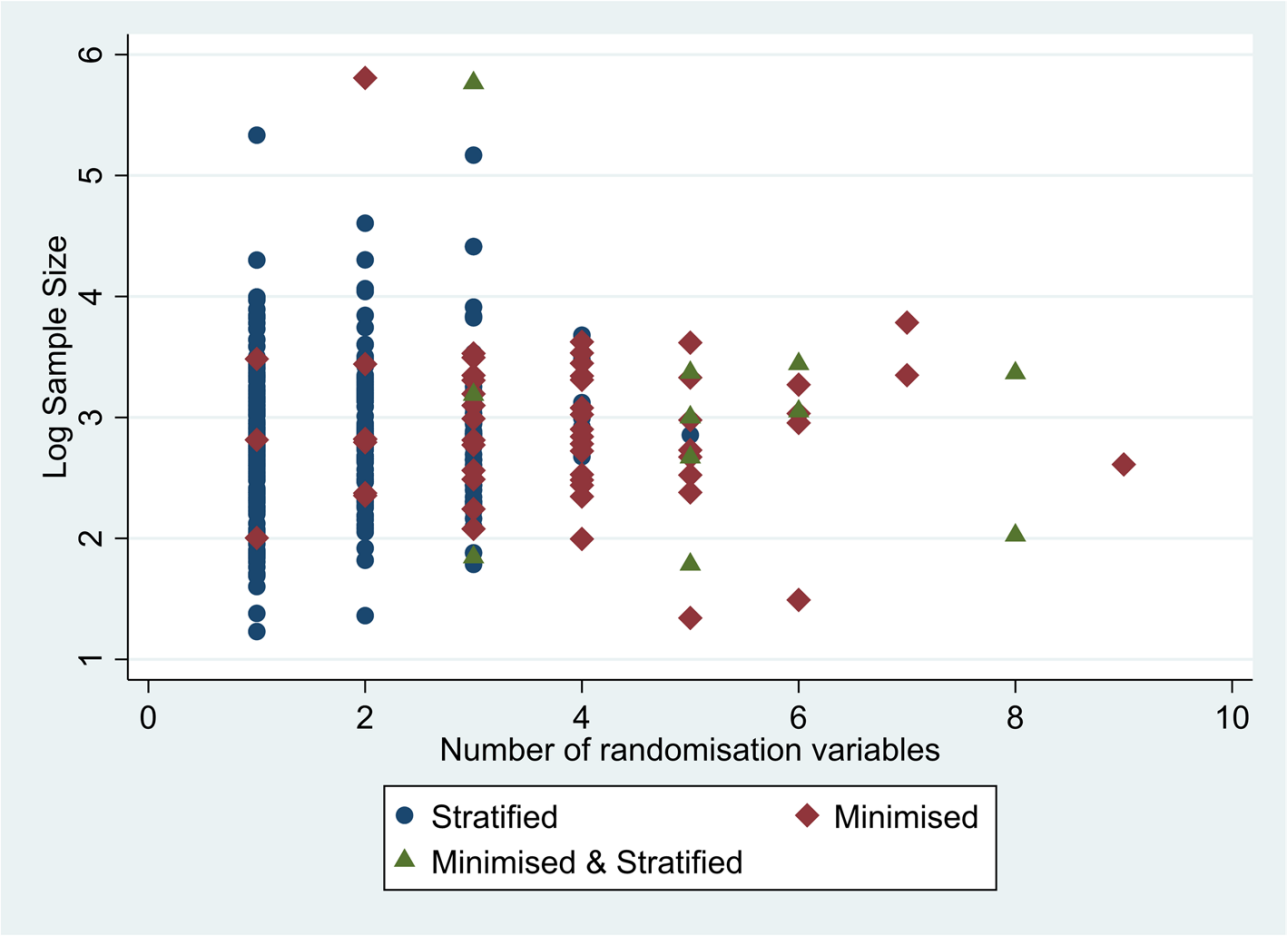
Plot of sample size vs the number of randomisation variables included. **Note**: y axis is presented as log base 10

**Fig. 3:**
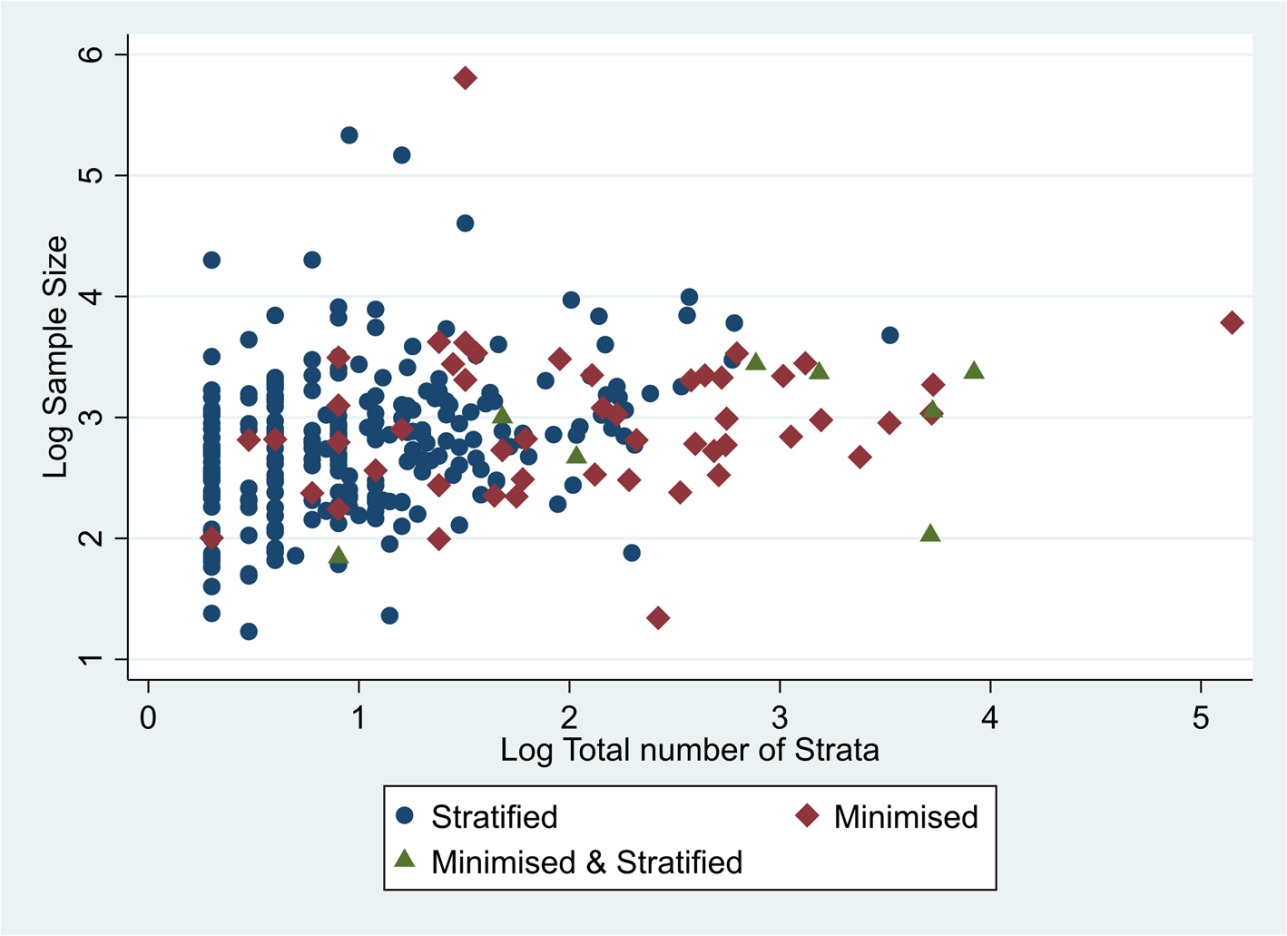
Plot of sample size vs the number of strata across all randomisation variables. **Note**: Both axis are presented as log base 10

In order to look more closely at the difference between variables included in stratified randomisation and minimisation, we compared sample size with the number of randomisation variables included (shown in Fig. 2), and then sample size with the number of strata (in this case defined to be the product of categories for these variables (Shown in Fig. 3)). Both Figs. 2 and 3 suggest that stratification is more common in higher sample sizes, but that minimisation is used more frequently for more complex designs, when there is a greater number of randomisation variables and strata.

This suggests that while a greater percentage of stratification variables had greater than five categories than minimisation variables, trials that used minimisation included a greater number of randomisation variables and so overall had a greater number of strata.

Table 5 presents a comparison of our results with those published by Ciolino et al. Here the original results from Ciolino are presented, alongside these results combined with the HTA journal and then our findings from 2019. Comparisons between 2014 and 2019 show two notable trends. In some ways trials seem to be more simplistic, with an increase in the number of trials that do not include a covariate within the randomisation, also seen in the increase in the use of simple randomisation. Nevertheless, of those trials that do include a covariate in the analysis, there is an increase in the number of covariates being used in the randomisation.

**Table 5:** A comparison of current practice with a review conducted in 2014

	**Study Characteristic**	**2014** **(*n* = 167)**^a^	**2014 with HTA** **(*n* = 196)**^a^	**2019** **(*n* = 385)**^a^
Cluster Randomised	No	160 (96)	184 (93)	348 (90)
	Yes	7 (4)	12 (7)	37 (10)
Covariates included in the allocation	No	15 (10)	21 (11)	73 (19)
	Yes	139 (90)	162 (89)	312 (81)
Number of covariates	1	53 (38)	63 (38)	121 (31)
	2	54 (39)	57 (34)	86 (23)
	3	23 (17)	25 (15)	61 (16)
	4	6 (4)	8 (5)	24 (6)
	5 +	3 (2)	14 (8)	93 (24)
Number of Centres	Single Centre	9 (5)	18 (9)	67 (17)
	Multicentre	158 (95)	178 (91)	318 (83)
Number of Arms	2	129 (77)	149 (76)	319 (83)
	3	16 (10)	23 (12)	45 (12)
	4	14 (8)	16 (9)	15 (4)
	5 +	8 (5)	8 (4)	6 (1)
Allocation Method	Purely Random	1 (1)	2 (1)	25 (6)
	Blocked	15 (10)	20 (11)	35 (9)
	Stratified or Stratified Block	123 (79)	135 (74)	244 (64)
	Minimisation/Covariate adaptive	14 (9)	23 (13)	57 (15)
	Other	2 (1)	3 (1)	24 (6)

Unless otherwise specified, the table reports the number of studies along with column percentages

^a^ Percentages are based on non-missing values

Finally, a comparison of our findings alongside the review from Kahan et al., is shown in Table 6. This shows an overall increase in the use of prognostic factors in the randomisation process, and more importantly, an increase in adjustment for balancing factors in subsequent analysis. Exploring the relationship between the number of centres, sample size, and subsequent adjustment for centre in the final analysis, we found that overall, random effects were more commonly used to adjust for centre and fixed effects were generally used in studies with less than 15 sites (shown in Additional file 1: Appendix Fig. 1).

**Table 6:** A comparison with a previous review of the inclusion of randomisation factors in the analysis

Study characteristic	Kahan review (2010)	Our results (2019)
Total Trials using at least one balancing factor	41	247
Centre as balancing factor	35 / 41 (85%)	80 / 247 (32%)
At least one patient level prognostic factor	24 / 41 (59%)	189 / 247 (77%)
Adjusted for All balancing variables in analysis	14 / 41 (34%)	147 / 247 (60%)
Balanced on centre and adjusted for centre	13 / 35 (37%)	33 / 80 (41%)
Balanced on prognostic factor and adjusted for all in analysis	7 / 24 (29%)	145 / 189 (77%)

Unless otherwise specified, the table reports the number of studies along with column percentages

## Discussion

This review gives an insight into the randomisation methods most commonly used and shows that there have been very few changes in this methodology since 2014, with a method containing an element of stratification being favoured. Response adaptive randomisation methods seem to be less commonly used, but this may be due to the types of trials being conducted, as these methods are only appropriate in very specific circumstances which may be less likely to be published in the journals we have reviewed.

We expected to see trials with larger sample sizes using less complex methods, as achieving balance is more likely to happen without intervention in larger sample sizes; however we observed the opposite to be true. It is possible that researchers may have been reluctant to use more complex methods with smaller sample sizes, as a large number of strata with a small sample size can lead to overstratification [8]; however this is perhaps surprising as these methods were devised to create more balanced groups with smaller sample sizes.

European Medicines Agency (EMA) guidance on covariate adjustment was issued in 2015 [9], so was not available during the 2014 review conducted by Ciolino but was available during our review. The EMA discuss the benefits of stratification for improving estimation efficiency. It also states that all stratification variables should be included within subsequent analysis unless they were chosen only for administrative reasons, and in this case justification for non-inclusion should be given. Their views on dynamic methods are more cautious as they state the possibility of issues with type 1 error control when using these methods.

When discussing inclusion on centre in an analysis, the EMA recommends all variables should be included in the analysis but recognises there are many issues leading to unreliable estimates, and states that in the case of non-inclusion of centre this should be explained, and sensitivity analysis should be used to demonstrate there is no effect of leaving it out of the model.

ICH E9 recommends that inclusions of centre should be dependent on the effect of centre, and discusses situations in which inclusions of centre in subsequent analysis may not be practical, such as those with a large number of smaller centres that recruit a limited number of participants [10].

We noted that trials which had stratified by centre tended not to include centre in subsequent analysis. This may allude to a more practical issue where models including centre can be a lot more unstable if there are a large number of centres and some centres with small recruitment numbers [11].

Looking at trends in methods use over time, we were surprised to see that fewer studies included a covariate in the analysis, but those that did were including a larger number of covariates than previously seen in 2014. This may suggest a polarisation of opinions, with some researchers sticking to less complex methods without inclusion of covariates, whilst those that do include covariates assume a benefit in including larger numbers of them. Additionally, generally we can see those studies including less than 5 variables in their randomisation are much more likely to use stratification, where above this minimisation becomes more common, which is consistent with existing guidance [12].

Our findings agree with McPherson’s [5], that the preferred method of randomisation is block stratified randomisation. Overall, the only design characteristics that seem to factor into researchers’ decisions on which randomisation method they select is related to the sample size and the number of centres, creating a need for more in-depth research into how these decisions are actually made.

Comparing our findings to those from Kahan et al., there is a large increase in trials adjusting for balancing factors in subsequent analysis, suggesting a large influence of this research in practice. When including centre in the analysis, this may be included in multiple different ways, as discussed by Senn [13]. In our review, more studies adjusted for site in their analysis after including site in the randomisation than Kahan found in 2010, although almost half of the studies did not adequately specify how site was included (as a random or fixed effect). There does however seemed to be a correlation between the number of centres a trial has and whether a random or fixed effect was used.

This study benefits from being a large and comprehensive review. The four clinical journals were selected as they were used in many other reviews, allowing this review to update previously published research but also as they are considered high impact journals, and since each journal is committed to the CONSORT statement [14], it was felt they would provide more complete data.

The generalisability of results may be considered a limitation of this review as it only focuses on five high impact journals. The addition of the NIHR HTA library was intended to widen the scope of journals from the other 4 clinical journals, but we do still acknowledge that the papers reviewed will still have limited generalisability to all clinical trials.

Another limitation of this review is that results may be considered slightly out of date. However, due to the COVID-19 pandemic, we felt that publications after this year would not be representative of usual practice, hence we felt that 2019 gave the most up to date representative review of the literature.

The findings of this review demonstrate that there is some evidence of trial design characteristics contributing to a researcher’s choice of randomisation method. There has not been much evolution in randomisation methodology, with simple, block, stratified and minimisation making up 95% of individually randomised trials in this review, suggesting little uptake in any newer emerging randomisation methods. The one noticeable change in practice is the polarisation of method use, which adds evidence to the fact the differing opinions on randomisation method affects methods use in clinical trials. Future research will focus on how study features factor into a researcher’s decision when choosing a randomisation method, and other factors that may affect their choice.

## Supplementary Information

**Additional file 1:** **Appendix Graph 1.** Scatter plot showing the number of centres vs the sample size.

**Additional file 2: Appendix Table 1. **A summary of the data extraction variables.

**Additional file 3:** **Appendix Table 2.** A summary of the criteria for disagreements and discrepancies.

**Additional file 4:** **Appendix Table 3. **A summary table showing the main study characteristics of 2019 papers split by randomisation method.

## Data Availability

Project data are available upon request, subject to the NCTU data sharing policy. Please contact the corresponding author to request data.
