# Supplementary material for: A systematic review of randomisation method use in RCTs and association of trial design characteristics with method selection
Source: BMC Med Res Methodol. 2022 Dec 7;22:314. doi: 10.1186/s12874-022-01786-4 (PMC9727841; doi:10.1186/s12874-022-01786-4)
Supplement: Supplementary file 1 — Additional file 1: Appendix Graph 1. Scatter plot showing the number of centres vs the sample size. [file 12874_2022_1786_MOESM1_ESM.docx]

*Appendix Graph 1: Scatter plot showing the number of centres vs the sample size*

**

*Scatter plot is shown for trials that included centre as one of their randomisation strata, subsequently included centre in their analysis and explicitly stated how centre was included. Of the 33 trials which adjusted for centre after inclusion in the randomisation, 12 reported using a random effect, 4 a fixed effect, and the other 17 did not adequately specify which method was used.*
