# Supplementary material for: A systematic review of randomisation method use in RCTs and association of trial design characteristics with method selection
Source: BMC Med Res Methodol. 2022 Dec 7;22:314. doi: 10.1186/s12874-022-01786-4 (PMC9727841; doi:10.1186/s12874-022-01786-4)
Supplement: Supplementary file 2 — Additional file 2: Appendix Table 1. A summary of the data extraction variables. [file 12874_2022_1786_MOESM2_ESM.docx]

*Appendix Table 1 – A summary of the data extraction variables.*

| **Variable** | **Description** |
| --- | --- |
| Author | First author recorded |
| Study start year | The year recruitment began |
| Sample size | The final size of the study |
| Specialisation | Defined using the UKCRC Health Research Classification System |
| Reference |  |
| Protocol needed | Yes/No indicator if a source other than the paper was needed to fill out this section |
|  |  |
| Randomisation | Tick boxes used to record each method that was used of: Simple, Block, Stratification, Minimisation, Bayesian or other |
| Other Please Specify | Specification and details were given if other |
| Other details | Free text |
| Randomisation details | A free text box used to collect all of the information on the text on randomisation method for clarification |
| Protocol needed | Yes/No indicator if a source other than the paper was needed to fill out this section |
|  |  |
| Blinding method | Tick boxes used to record each type that was included of: Patient, Clinician, Outcome Assessor, Statistician, None or Unknown |
| Protocol needed | Yes/No indicator if a source other than the paper was needed to fill out this section |
|  |  |
|  |  |
| Multi centre | Whether the trial was multicentre or not an if so, how many centres. |
| Multi-arm | Whether the trial was multiarm or not and if so, how many arms were included. |
| Cluster | Whether the trial was cluster randomised or not and if so, how many clusters were included. |
| Factorial Design | Whether a factorial design was used |
| Crossover Design | Whether a crossover design was used |
| Matching | Whether matching was used in the randomisation |
| Additional Information | A free text box used to collect any other information relating to design |
| Protocol needed | Yes/No indicator if a source other than the paper was needed to fill out this section |
|  |  |
| Variables in randomisation | Whether a variable was included within the randomisation and if so, how many were included/ |
| Protocol needed | Yes/No indicator if a source other than the paper was needed to fill out this section |
|  |  |
| Randomisation variable information | For each randomisation variable included:  Whether the variable was stratified on or minimised  Whether the variable was binary, categorial or continuous.  If the variable was categorical, was it ordinal.  If the variable was continuous, how was it split.  The number of categories of the variable  Whether it was included in the analysis and if so what form it was included as. |
