# Supplementary material for: A systematic review of randomisation method use in RCTs and association of trial design characteristics with method selection
Source: BMC Med Res Methodol. 2022 Dec 7;22:314. doi: 10.1186/s12874-022-01786-4 (PMC9727841; doi:10.1186/s12874-022-01786-4)
Supplement: Supplementary file 3 — Additional file 3: Appendix Table 2. A summary of the criteria for disagreements and discrepancies. [file 12874_2022_1786_MOESM3_ESM.docx]

*Appendix Table 2 – A summary of the criteria for disagreements and discrepancies.*

| A disagreement was defined as when the two extractions contained contradictory information.  Disagreements were identified as: | - Difference in study start year. - Difference in sample size. - Differences in study characteristics (multicentre, multiarm or cluster randomised). - Differences in the number of centres, arms, or clusters. - Differences in study characteristics (factorial, crossover, or matching). - Differences in the number of variables included in the randomisation. - Differences in the number of categories in a randomisation variable. |
| --- | --- |
| A discrepancy defined any difference between the two extractions. Including the definition of disagreement but also differences between the two extractions that are not contradictory.  Additional items defined as discrepancies were identified as: | - Differences in disease specification (Sometimes more than one may be true) - Differences in the classification of randomisation method (as simple block randomisation is equivalent to block randomisation) - Differences in blinding status (As information in papers is sparce so in some cases this is due to interpretation) - Differences in randomisation variable classification (Some variables were categorical but split as binary) - Differences in additional design information (As this information may be recorded in a different part of the form) |
