# Supplementary material for: A systematic review of randomisation method use in RCTs and association of trial design characteristics with method selection
Source: BMC Med Res Methodol. 2022 Dec 7;22:314. doi: 10.1186/s12874-022-01786-4 (PMC9727841; doi:10.1186/s12874-022-01786-4)
Supplement: Supplementary file 4 — Additional file 4: Appendix Table 3. A summary table showing the main study characteristics of 2019 papers split by randomisation method. [file 12874_2022_1786_MOESM4_ESM.docx]

*Appendix Table 3 – A summary table showing the main study characteristics of 2019 papers split by randomisation method.*

| **Study Characteristic** | | **Total**  **[n (%)]** | **Simple** **[n (%)]** | **Block**  **[n (%)]** | **Stratified**  **[n (%)]** | **Stratified (using centre)**  **[n (%)]** | **Block Stratified**  **[n (%)]** | **Block stratified (using centre)**  **[n (%)]** | **Minimisation**  **[n (%)]** | **Minimisation (using centre)**  **[n (%)]** |
| --- | --- | --- | --- | --- | --- | --- | --- | --- | --- | --- |
| Unit of Allocation | Individual | 348 (90) |  |  |  |  |  |  |  |  |
|  | Cluster | 37 (10) |  |  |  |  |  |  |  |  |
| If Cluster, | 2-25 | 15 (40) |  |  |  |  |  |  |  |  |
| Number of Clusters | 26-50 | 8 (22) |  |  |  |  |  |  |  |  |
|  | 51+ | 14 (38) |  |  |  |  |  |  |  |  |
| **For individually randomised studies**^1^ | | | | | | | | | | |
|  |  | **(n = 330)** | **(n = 20)** | **(n = 33)** | **(n = 48)** | **(n = 18)** | **(n = 94)** | **(n = 14)** | **(n = 19)** | **(n = 30)** |
| Number of Arms | 2 | 270 (82) | 16 (80) | 30 (91) | 34 (71) | 17 (94) | 45 (66) | 83 (88) | 16 (84) | 29 (97) |
|  | 3 | 39 (12) | 3 (15) | 1 (3) | 13 (27) | 1 (6) | 10 (15) | 8 (9) | 3 (6) | 0 |
|  | 4 | 15 (4) | 0 | 1 (3) | 1 (2) | 0 | 9 (13) | 3 (3) | 0 | 1 (3) |
|  | 5+ | 6 (2) | 1 (5) | 1 (3) | 0 | 0 | 4 (6) | 0 | 0 | 0 |
| Number of Centres ^2^ | Single Centre | 27 (8) | 4 (20) | 5 (15) | 4 (8) | 0 | 13 (19) | 0 | 1 (2) | 0 |
|  | 2-10 | 81 (25) | 4 (20) | 11 (34) | 3 (6) | 7 (39) | 11 (16) | 40 (43) | 2 (11) | 3 (10) |
|  | 11-25 | 65 (20) | 7 (35) | 4 (12) | 2 (4) | 2 (11) | 9 (14) | 31 (34) | 3 (16) | 7 (23) |
|  | 26-50 | 45 (14) | 1 (5) | 4 (12) | 10 (21) | 3 (17) | 6 (9) | 7 (8) | 4 (21) | 10 (33) |
|  | 51-100 | 39 (12) | 1 (5) | 4 (12) | 10 (21) | 1 (5) | 9 (14) | 6 (6) | 3 (16) | 5 (17) |
|  | 101+ | 70 (21) | 3 (15) | 5 (15) | 19 (40) | 5 (28) | 19 (28) | 8 (9) | 6 (32) | 5 (17) |
|  | **Median (IQR)** | **22 (6, 86)** | **15 (4, 29)** | **11 (4, 51)** | **70 (29, 160)** | **22 (7, 102)** | **28 (2, 113)** | **13 (6, 23)** | **48 (13, 73)** | **35 (14, 73)** |
|  | **Mean (sd)** | **71 (141)** | **59 (127)** | **121 (324)** | **117 (136)** | **94 (142)** | **76 (121)** | **33 (71)** | **72 (74)** | **56 (57)** |
| Size of Study | < 200 | 60 (18) | 6 (30) | 10 (30) | 12 (15) | 3 (17) | 9 (13) | 14 (15) | 4 (21) | 1 (3) |
|  | 201-1000 | 159 (48) | 9 (45) | 15 (46) | 20 (42) | 6 (33) | 39 (58) | 47 (50) | 8 (42) | 16 (53) |
|  | 1001-10,000 | 103 (31) | 4 (20) | 6 (18) | 15 (31) | 9 (50) | 17 (25) | 32 (34) | 7 (37) | 13 (43) |
|  | 10,001+ | 8 (3) | 1 (5) | 2 (6) | 1 (2) | 0 | 3 (4) | 1 (1) | 0 | 0 |
|  | **Median (IQR)** | **648 (240, 1487)** | **354 (133, 1445)** | **417 (154, 782)** | **652 (209, 1460)** | **905 (475, 4786)** | **579 (304, 1217)** | **680 (247, 1435)** | **653 (237, 2236)** | **927 (528, 2135)** |
|  | **Mean (sd)** | **3343 (31370)** | **1493 (2910)** | **1845 (3980)** | **1453 (3083)** | **2889 (3626)** | **2042 (4972)** | **1535 (4291)** | **1322 (1437)** | **1361 (1304)** |
| Blinding | No | 194 (59) | 9 (45) | 18 (55) | 8 (17) | 11 (61) | 44 (65) | 69 (73) | 13 (68) | 22 (73) |
|  | Yes | 136 (41) | 11 (55) | 15 (45) | 40 (83) | 7 (39) | 24 (35) | 25 (27) | 6 (32) | 8 (27) |

Unless otherwise specified, the table reports the number of studies along with column percentages.

^1^ 6 trials that used stratified minimisation and 12 defined as other are not included.

^2^ 3 trials that used block stratification did not report the number of centres
